# Neurocognitive function across different phases of bipolar disorder: an evaluation using the B-CATS

**DOI:** 10.3389/fpsyt.2025.1590198

**Published:** 2025-05-08

**Authors:** Lu Tian, Yi Liu, Jinjie Xu, Zhen Mao, Xiaomeng Xing, Qijing Bo, Chen Hu

**Affiliations:** Beijing Key Laboratory of Mental Disorders, National Clinical Research Center for Mental Disorders & National Center for Mental Disorders, Beijing Anding Hospital, Capital Medical University, Beijing, China

**Keywords:** bipolar disorder, neurocognitive function, brief cognitive assessment tool for schizophrenia, depression, manic, euthymic

## Abstract

**Objectives:**

Neurocognitive dysfunction is a critical aspect of bipolar disorder (BD) and affected by multiple factors, which may serve as potential points for prevention and clinical intervention. This study aimed to compare the neurocognitive profiles of BD patients across different phases with those of healthy controls (HCs) via the Brief Cognitive Assessment Tool for Schizophrenia (B-CATS) and explore the relationship between neurocognitive impairment and mood symptom severity, advancing the applicability of the B-CATS for BD patients.

**Methods:**

This cross-sectional study included 238 BD patients, of whom 80, 78, and 80 were in the depressive, manic/hypomanic, and euthymic phases, respectively, and 80 HCs. The participants’ neurocognitive profiles were evaluated using the B-CATS, which includes 3 tests: the Animal Fluency Test (AFT), the Digit Symbol Substitution Test (DSST), and the Trail Making Test (TMT). In addition, the 17-item Hamilton Depression Rating Scale (HAMD-17), Hamilton Anxiety Rating Scale (HAMA), Young Mania Rating Scale (YMRS) and Sheehan Disability Scale (SDS) were used to assess depression symptoms, anxiety, mania, and the degree of disability.

**Results:**

Among the four groups, the depressive group showed the most severe neurocognitive impairment, followed by the manic group, and the euthymic group was inferior to that of the healthy control group (*p* < 0.001). Correlation analysis showed that in the depression group, depressive symptoms were inversely associated with AFT (*r* = -0.427, *p* < 0.001), DSST (*r* = -0.242, *p* = 0.030), and total scores (*r* = -0.248, *p* = 0.026). In the manic group, manic symptoms were inversely associated with B-CATS scores (*r* = -0.407, *p* < 0.001), and patients with current medication use had lower B-CATS total scores and TMT scores (*r* = -0.310, *p* = 0.006; *r* = -0.292, *p* = 0.010, respectively). Multiple regression analysis showed that B-CATS total score was closely related to SDS- Social life (p = 0.030) in depression, YMRS score (*p* < 0.001) and drugs (*p* < 0.001) in manic.

**Conclusions:**

Neurocognitive impairment in BD patients are present throughout the entire illness course, with the most severe deficits occurring during the depressive phase. B-CATS is a quick and simple tool for assessing neurocognitive function for BD patients.

## 1 Introduction

Bipolar disorder (BD) is a mental disorder characterized by a complex and varied clinical presentation that affects more than 2% of the global population and causes disability worldwide (1, 2). BD involves not only mood fluctuations, alternating or mixed manic/hypomanic episodes and depressive episodes but also frequent comorbidities such as psychotic symptoms, anxiety, obsessive-compulsive disorder, and substance abuse (3). Over the past few decades, neurocognitive impairment has been increasingly recognized as a core feature of BD that is present in both the acute phase and the remitted state (4–6). It is well documented that working memory, speed of information processing, coordination, executive function, and attention are particularly impacted in BD (7–9). Processing speed impairments have been proposed as a potential endophenotype of the disorder (10). Unlike episodic mood disturbances, neurocognitive dysfunction in BD patients is conceptualized as a trait-like feature of the disorder, with significant implications for long-term functioning and recovery (11–13). Even during euthymia, these impairments hinder goal-directed behavior, affect problem-solving abilities, and limit interpersonal functioning, thereby reducing overall quality of life (14). Moreover, these deficits are associated with lower treatment adherence, poorer functional recovery, and a greater risk of relapse (15).

As neurocognitive dysfunction is a critical aspect of psychiatric disorders and a treatment challenge, numerous neurocognitive assessment tools have been developed for clinical and research purposes. However, specific tools for evaluating neurocognitive function in BD patients are currently lacking. Rossetti and colleagues conducted a systematic review of the neuropsychological instruments for BD, revealing that the currently employed cognitive assessment tools appear to be sufficiently sensitive to differentiate between BD patients with and without cognitive impairment; however, an optimal tool has yet been identified (16). The MATRICS Consensus Cognitive Battery (MCCB), developed by the National Institute of Mental Health (NIMH) in 2003, is a widely used tool in psychiatry that comprises seven domains and ten subtests (17). The International Society for Bipolar Disorders (ISBD) Consensus on Neurocognitive Assessment Tools recognizes the MCCB as an excellent starting point for assessing neurocognitive deficits in BD research (18). Numerous studies have applied the MCCB to evaluate neurocognitive function in BD patients (19), but its complexity and time consumption limit its practical use.

In 2011, Hurford et al. developed a brief version of the MCCB, the Brief Cognitive Assessment Tool for Schizophrenia (B-CATS), which provides a quick overall score reflecting a patient’s neurocognitive function (20). Including the Animal Fluency Test (AFT), the Digit Symbol Substitution Test (DSST), and the Trail Making Test (TMT), the B-CATS measures critical domains of neurocognitive functioning, including processing speed, verbal memory, and working memory, through tasks that can be completed in under 10 minutes. Subsequently, Hurford et al (21) confirmed that the B-CATS exhibits robust psychometric properties, demonstrating strong test-retest reliability and satisfactory internal consistency, and practice effects are small. It correlates 0.76 (p<0.01) with the MCCB, and its reliabilities were moderate (α=0.6) (22). Specifically, the main neurocognitive impairments in BD patients are executive function, working memory, verbal memory and attention impairments, with evidence suggesting that these impairments may worsen during mood episodes but remain detectable even in euthymia (23–26). As brief cognitive screeners and comprehensive batteries may be appropriate for detecting or monitoring cognitive changes in BD, the B-CATS may serve as a potential method for the rapid and accurate assessment of neurocognitive function in BD patients. However, to our knowledge, no study thus far has applied this tool to BD patients in China.

This study sought to evaluate neurocognitive performance in BD patients across different phases of the illness—manic, depressive, and euthymic phases via the B-CATS for the first time. Furthermore, this study aimed to explore the relationship between neurocognitive impairment and the severity of mood symptoms and deepen our understanding of the interplay between neurocognitive deficits and symptomatology in patients with BD. By addressing the gaps mentioned above, this study aimed to advance the applicability of the B-CATS for BD patients and provide a foundation for future clinical and research-based evaluations of neurocognitive function in this population.

## 2 Materials and methods

### 2.1 Participants

This was a cross-sectional study conducted at Beijing Anding Hospital, Capital Medical University, from July 2021 to June 2023. Patients in the acute phase were consecutively enrolled as they were admitted to the hospital, while those in the euthymic phase were randomly selected from a pool of eligible candidates using a computer-generated randomization list. Inclusion criteria were (1): aged between 18 and 60 years, with sufficient auditory and visual abilities to complete the study (2); had ≥9 years of formal education (3); met the diagnostic criteria for BD in the Diagnostic and Statistical Manual of Mental Disorders, Fifth Edition (DSM-5), with remission defined as a 17-item Hamilton Depression Rating Scale (HAMD-17) total score ≤7 points and a Young Mania Rating Scale (YMRS) total score ≤6 points (4); had not received modified electroconvulsive therapy (MECT) or repetitive transcranial magnetic stimulation (rTMS) treatment in the previous six months; and (5) had a signed informed consent form. The exclusion criteria were as follows (1): patients with secondary depressive/manic symptoms (physical diseases, drugs, or other mental diseases) (2); patients with a history of or current significant organic brain diseases or severe unstable physical diseases (3); pregnant and lactating women; and (4) patients with severe impulsive aggressive behavior or suicidal thoughts and suicide attempts. Healthy controls (HCs) from the community were enrolled through recruitment advertisements, completed an interview and met the following inclusion criteria (1): aged between 18 and 60 years (2); did not meet the DSM-V diagnostic criteria for any psychiatric disorder; and (3) had no related family history. The exclusion criteria for HCs were as follows (1): had a history of or current significant medical or neurological conditions (2); had a history of head trauma or unconsciousness lasting >1 hour (3); had a history of or current significant drug/alcohol abuse; and (4) were pregnant.

The clinical research ethics committees of Beijing Anding Hospital approved the study protocol. After complete description of the study to the subjects, a written informed consent was obtained. Each participant will receive an evaluation by a professional physician but will not be provided with any additional compensation.

### 2.2 Assessments

Each participant’s sociodemographic data, including age, gender, education level, occupation, marital status, and family history, were collected with a questionnaire designed for the study. All participants were diagnosed with bipolar disorder based on the criteria outlined in the Diagnostic and Statistical Manual of Mental Disorders, Fifth Edition (DSM-5) (27). The 17-item Hamilton Depression Rating Scale (HAMD-17) (28), Hamilton Anxiety Rating Scale (HAMA) (29) and Young Mania Rating Scale (YMRS) (30) were used to assess depressive, anxiety-related, and manic symptoms. Neurocognitive functioning was assessed with the B-CATS (20), which is extracted directly from the Chinese-adapted MCCB (31) and includes 3 tests: the Animal Fluency Test (AFT), the Digit Symbol Substitution Test (DSST), and the Trail Making Test (TMT). Overall, the B-CATS reflects the speed of information processing in an individual’s neurocognitive process. Furthermore, the Sheehan Disability Scale (SDS) was used to assess the degree of disability caused by psychological problems in individuals. The questionnaire was written in Mandarin Chinese and each question was scored using predefined categorical or numerical scales (e.g., age in years, marital status categorized as unmarried, married, divorce or widowed). The team collaboratively designed the questions to ensure clarity, relevance, and cultural appropriateness for the study population.

### 2.3 Procedure

In this study, after obtaining written informed consent and confirming eligibility, participants were escorted to a quiet room where trained psychiatrists administered the questionnaire. The psychiatrists asked all questions and recorded the responses on a standardized form. The questionnaire included clear, standardized instructions, and the psychiatrist provided participants with a detailed explanation of the process. They were also available to address any questions or concerns during the administration. Interrater reliability remained within acceptable limits.

The Verbal Fluency Test (VFT) assesses an individual’s language ability. The B-CATS only retains the AFT, which requires a subject to say as many animal names as possible within 1 minute, assessing language (semantic knowledge, naming, understanding), memory, executive speed, and other cognitive functions, which can more extensively reflect executive functions. The DSST requires individuals to match a unique geometric symbol with the corresponding Arabic numeral within a limited time of 90 seconds. The raw score is the number of correct items completed within the specified time limit. This test can assess an individual’s fine motor skills, executive speed, visual scanning ability, learning ability, and memory. The TMT is a neuropsychological test used to assess an individual’s visual attention and task switching ability. This test consists of parts A and B, and either part can be selected in the B-CATS assessment, requiring the individual to connect 25 dots as quickly as possible while maintaining accuracy. In this study, we choose to use Part A, which takes approximately 2 minutes to complete. Unlike the VFT and DSST scores, where higher scores indicate better neurocognitive function, higher TMT scores suggest poorer neurocognitive performance.

### 2.4 Statistical analysis

The data were entered into Epidata software version 3.1 and analyzed using SPSS 26.0 for Windows. Continuous variables are presented as means with standard deviations (SD), while categorical variables are expressed as frequencies and percentages. To maintain consistency and facilitate a more intuitive analysis of the results, the raw TMT scores were converted to negative values during the statistical analysis. One-way ANOVA was used to compare the demographic characteristics, clinical characteristics and neuropsychological test results among the four groups, and Bonferroni correction was used to measure multiple group differences. Analysis of covariance (ANCOVA) was performed to evaluate neurocognitive function and symptoms, adjusting for significant demographic variables. The chi-square test was used to analyze enumeration data among the four groups. Pearson’s or Spearman’s rank correlation analysis was used to measure the relationship between sociodemographic characteristics in each group. Multiple regression analysis was conducted to identify relationships between the B-CATS domain scores and relevant factors. For all analyses, the level of statistical significance was set at *p* < 0.05.

## 3 Results

### 3.1 Demographic and clinical characteristics of the participants

This study initially screened 274 potential participants, of whom 36 were excluded: 15 did not meet the diagnostic criteria for bipolar disorder (BD), 12 declined to participate, and 9 were excluded due to incomplete data. The final sample included 238 patients with BD, comprising 80 in the depressive (D) phase, 78 in the manic or hypomanic (M) phase, and 80 in the euthymic (E) phase, along with 80 healthy controls (HCs). The overall study sample had a mean age of 30.20 years (SD = 10.23), with a sex distribution of 48.7% male and 51.3% female. The mean age and standard deviation (SD) for each group were as follows: depressive patients (29.20 ± 11.27 years), manic or hypomanic patients (30.96 ± 10.37 years), euthymic patients (28.78 ± 10.66 years), and healthy controls (31.84 ± 8.63 years). The four groups showed no significant differences in age (*F* = 1.724, *p* = 0.164), sex (χ² = 0.300, *p* = 0.960), education level (*F* = 0.185, *p* = 0.906), or marital status (χ² = 8.179, *p* = 0.225). In addition, the three BD groups did not differ significantly in terms of duration of illness (*F* = 0.814, *p* = 0.444), age at illness onset (*F* = 3.082, *p* = 0.048), or attack times (*F* = 0.168, *p* = 0.845). In addition to the highest HAMD-17 score (*F* = 1369.754, *p* < 0.001, η² = 0.85) and the highest HAMA score (*F* = 232.390, *p* < 0.001, η² = 0.49) in the depression group, the euthymic group also had significantly higher HAMD-17 scores than did the HC group (*p* < 0.001, Cohen's *d* = 1.23). Manic or hypomanic patients had significantly higher YMRS scores than patients in the other three groups did (*F* = 720.426, *p* < 0.001, η² = 0.75), and there was no statistically significant difference in the YMRS score among the remaining three groups (*p* > 0.05). **Table 1** shows the demographic characteristics and the HAMD-17, HAMA, YMRS, SDS and B-CATS scores.

**Table 1:** Demographic characteristics and B-CATS scores.

Characteristics	Depressed (n=80)		Manic or hypomanic (n=78)		Euthymic (n=80)		Healthy controls (n=80)		ANOVA		*Post Hoc* Analysis
	Mean	SD	Mean	SD	Mean	SD	Mean	SD	*F*	*P*	
Age (years)	29.20	11.27	30.96	10.37	28.78	10.66	31.84	8.63	1.724	0.164	–
Education level (years)	14.13	2.67	14.24	2.55	14.15	2.27	14.39	2.37	0.185	0.906	–
Duration of illness (months)	108.68	83.24	91.97	81.83	98.76	83.36	–	–	0.814	0.444	–
Age at illness onset	20.20	7.99	23.36	8.77	20.91	8.35	–	–	3.082	0.048	–
Attack times	4.01	2.06	3.94	2.60	4.16	2.46	–	–	0.168	0.845	–
Duration of the current episode ^a^	12.39	10.82	5.22	9.71					44.083	<0.001	M< D
HAMD total score	28.18	3.80	0.79	0.99	2.51	1.74	0.45	0.83	1369.754	<0.001	M, C< E< D
HAMA total score	22.33	7.47	0.71	1.31	1.18	1.48	0.31	0.69	232.390	<0.001	M, E, C< D
YMRS total score	0.28	0.67	24.04	4.54	0.35	0.78	0.05	0.22	720.426	<0.001	D, E, C< M
B-CATS total score	-5.11	26.98	15.74	29.02	34.66	22.94	55.06	13.02	93.869	<0.001	D< M< E< C
AFT	15.36	3.73	17.76	3.81	19.74	3.68	24.68	2.92	117.811	<0.001	D< M< E< C
DSST	34.45	10.18	38.08	13.37	47.91	13.64	56.63	6.99	101.366	<0.001	D, M< E< C
-TMT	-54.92	16.62	-40.09	17.09	-32.99	10.67	-26.24	5.90	67.664	<0.001	D< M< E< C
SDS											
Work/study	7.68	1.39	6.49	2.24	3.00	1.68	–	–	145.295	<0.001	E< M< D
Social life	7.39	1.68	5.86	2.62	2.59	1.71	–	–	115.059	<0.001	E< M< D
Family life	6.96	1.63	6.10	2.20	2.61	1.52	–	–	129.836	<0.001	E< M< D
	*N*	*%*	*N*	*%*	*N*	*%*	*N*	*%*	*χ^2^*	*P*	
Sex (male) ^b^	39	48.75	40	51.28	38	47.50	38	47.50	0.300	0.960	–
Married ^b^	24	30.00	33	42.31	28	35.00	37	46.25	8.179	0.225	–
Employed ^b^	27	33.75	38	48.72	34	42.50	62	77.50	45.678	<0.001	D, M, E< C
Family history(yes)^b^	8	10.00	11	14.10	11	13.75	–	–	0.747	0.668	–
Type I ^b^	25	31.3	61	78.2	38	47.5	–	–	35.914	<0.001	D, E < M
Current use of drugs ^b^	41	51.25	31	39.74	52	65.00	–	–	14.567	0.001	M < E

^a^ T test. ^b^ χ^2^ analysis. All other values result from analysis of variance, homogeneity of variance with Bonferroni correction and heterogeneity of variance with Tamhane’s correction for *post hoc* tests. D, depression; M, manic or hypomanic; E, euthymic; C, health control; HAMD, The 17-item Hamilton Depression Rating Scale; HAMA, Hamilton Anxiety Rating Scale; YMRS, Young Mania Rating Scale; SDS, the Sheehan Disability Scale; B-CATS, the Brief Cognitive Assessment Tool for Schizophrenia. AFT, Animal Fluency Test; DSST, Digit Symbol Substitution Test; TMT, Trail Making Test.

### 3.2 Comparison of neurocognitive function among BD patients in the depressive, manic or hypomanic, and euthymic phases and HCs

**Figure 1:**
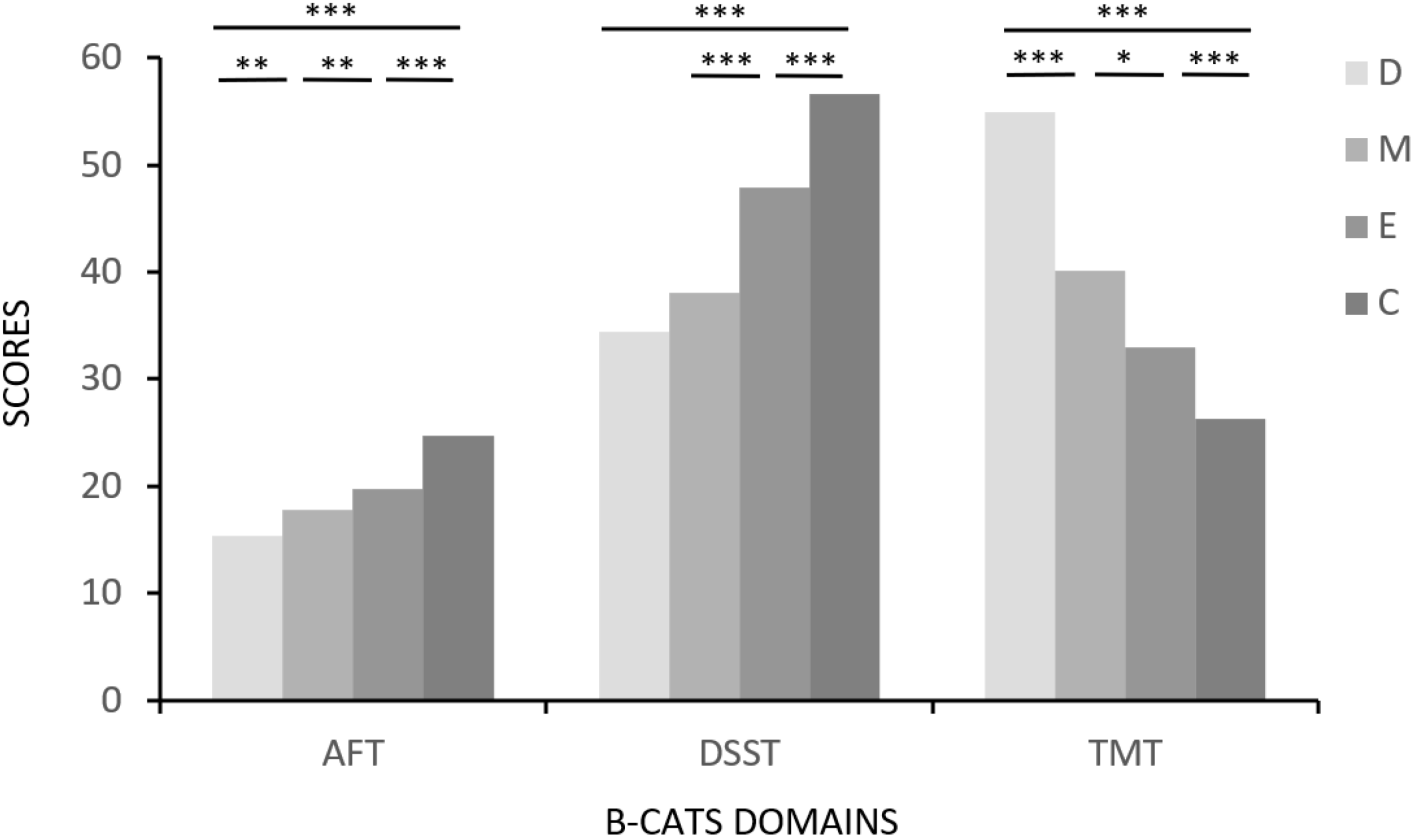
Comparison of B-CATS domain scores between bipolar disorder patients across different phases and healthy controls. D, depression; M, manic or hypomanic; E, euthymic; C, health control; AFT, Animal Fluency Test; DSST, Digit Symbol Substitution Test; TMT, Trail Making Test. ⁎ < 0.05 (2-tailed) ⁎⁎ < 0.01 (2-tailed) ⁎⁎⁎ < 0.001 (2-tailed).

Patients with BD performed worse in all B-CATS domains. Specifically, patients with depression obtained significantly lower AFT (*F* = 117.811, *p* < 0.001, η² = 0.33), TMT (*F* = 67.664, *p* < 0.001, η² = 0.22), and total scores (*F* = 93.869, *p* < 0.001, η² = 0.28) than the other three groups, whereas the DSST scores, although lower than those of the HC and euthymic groups, were not significantly different from those of the manic or hypomanic group (*F* = 101.366, *p* < 0.001, η² = 0.30). Compared with the euthymic and HC groups, the manic or hypomanic group had lower scores in all the B-CATS domains. Patients in the euthymic phase did not fare much better, obtaining worse scores in all neurocognitive domains than HCs did. The B-CATS demonstrated large effect sizes (Cohen’s *d* = -2.96, -1.82, -1.03 respectively) in differentiating bipolar disorder patients (depressive, manic, euthymic phases) from healthy controls, with the most pronounced impairment in the depressive phase. Inter-group comparisons revealed significant cognitive distinctions, particularly between depressive and euthymic phases (Cohen’s *d* = -1.35, *95% CI* = [-1.77, -0.93]), underscoring B-CATS’ sensitivity to phase-specific deficits and residual impairment in remission. The Cronbach's alpha of the B-CATS was 0.77 within our sample, which was acceptable. **Figure 1** presents the differences in the B-CATS domain scores among the groups. **Table 2** shows comparisons of neurocognitive impairment across BD and health controls and the discrimination ability of B-CATS for different stages.

**Table 2:** Statistical comparisons of cognitive impairment across bipolar disorder phases using B-CATS total score.

Comparison	*t*	*p*	*Cohen’s d [95% CI]*
DE vs. HC	-13.72	<0.001	-2.96 [-3.48, -2.44]
MA vs. HC	-8.16	<0.001	-1.82 [-2.29, -1.35]
EU vs. HC	-4.46	<0.001	-1.03 [-1.46, -0.60]
DE vs. MA	-4.04	<0.001	-0.81 [-1.21, -0.41]
DE vs. EU	-7.06	<0.001	-1.35 [-1.77, -0.93]
MA vs. EU	-3.01	0.003	-0.50 [-0.83, -0.17]

DE, depression; MA, bipolar disorder; EU, euthymic; HC, health control; Cohen’s d thresholds, Small (|*d*| ≥0.2), Medium (|*d*| ≥0.5), Large (|*d*| ≥0.8).

### 3.3 Associations between demographic and clinical characteristics and B-CATS composite scores in patients with BD and HCs

Depressive symptoms were inversely associated with AFT (*r* = -0.427, *p* < 0.001), DSST (*r* = -0.242, *p* = 0.030), and total scores (*r* = -0.248, *p* = 0.026) in the depression group. These findings suggest that more severe depressive symptoms are linked to poorer performance in attention, processing speed, and overall cognitive functioning. BD type was associated with neurocognitive function in the depression group, and patients with BD type I presented worse neurocognitive functioning (*r* = -0.349, *p* = 0.002), indicating that BD subtype may play a role in the severity of cognitive impairment. Furthermore, age, age at illness onset, length of illness, and attack frequency were negatively correlated with scores across all B-CATS domains, whereas employment status was positively correlated with B-CATS domain scores, except for the DSST score (**Table 3**). These results highlight the potential impact of illness chronicity and psychosocial factors on cognitive functioning in patients with BD.

In the manic or hypomanic group, manic symptoms were inversely associated with B-CATS domain scores and total scores (*r* = -0.561, *p* < 0.001; *r* = -0.394, *p* < 0.001; *r* = -0.257, *p* = 0.023; and *r* = -0.407, *p* < 0.001, respectively). This suggests that more severe manic symptoms may exacerbate cognitive deficits, particularly in executive functioning and attention. Age, education level, and the use of psychiatric medications were associated with TMT scores and total scores. Patients who were currently taking medication obtained lower B-CATS total scores and TMT scores (*r* = -0.310, *p* = 0.006; *r* = -0.292, *p* = 0.010; respectively), indicating poorer neurocognitive functioning (**Table 3**). These findings may reflect the cognitive side effects of certain medications or the severity of illness in patients requiring pharmacotherapy.

For patients in remission, clinical factors such as depressive, anxious, and manic symptoms were associated with neurocognitive functioning, but these associations were not statistically significant. Age, education level, age at illness onset, and duration of illness were significantly associated with the B-CATS domain scores and total score (**Table 3**). These results suggest that even in remission, demographic and illness-related factors continue to influence cognitive performance, underscoring the persistent nature of cognitive deficits in BD.

**Table 3:** Correlations between sociodemographic characteristics, clinical characteristics, and B-CATS domain scores of BD patients across different phases.

	Depressed (n = 80, *r*)				Manic or hypomanic (n = 78, *r*)				Euthymic (n = 80, *r*)			
	AFT	DSST	-TMT	Total	AFT	DSST	-TMT	Total	AFT	DSST	-TMT	Total
Age	-0.647^**^	-0.419^**^	-0.692^**^	-0.674^**^	-0.164	-0.170	-0.388^**^	-0.329^**^	-0.510^**^	-0.430^**^	-0.460^**^	-0.551^**^
Sex	0.053	0.145	0.212	0.159	0.028	-0.009	0.17	0.080	0.066	-0.078	0.023	-0.038
Education level	-0.216	0.000	-0.120	-0.104	-0.054	-0.203	-0.246^*^	-0.245^*^	0.164	0.070	0.261^*^	0.190
Marital status	-0.460^**^	-0.445^**^	-0.562^**^	-0.568^**^	-0.064	-0.243^*^	-0.272^*^	-0.288^*^	-0.455^**^	-0.403^**^	-0.188	-0.424^**^
Employed	0.241^*^	0.174	0.310^**^	0.291^**^	0.099	0.094	0.198	0.161	0.138	0.166	0.087	0.171
BD type	0.349^**^	0.379^**^	0.370^**^	0.408^**^	0.159	0.190	0.161	0.201	0.183	0.089	0.148	0.164
Age at illness onset	-0.423^**^	-0.371^**^	-0.640^**^	-0.593^**^	-0.117	-0.074	-0.221	-0.180	-0.415^**^	-0.395^**^	-0.378^**^	0.477^**^
Duration of illness	-0.550^**^	-0.287^**^	-0.429^**^	-0.449^**^	-0.131	-0.175	-0.263^*^	-0.253^*^	-0.299^**^	-0.189	-0.260^*^	-0.281^*^
Attack times	-0.452^**^	-0.380^**^	-0.478^*^	-0.500^**^	-0.048	0.037	-0.086	-0.040	-0.093	0.078	-0.031	0.017
Duration of the current episode	0.122	0.175	0.050	0.114	0.082	0.152	0.168	0.180	–	–	–	–
Current use of drugs	0.057	-0.112	-0.129	-0.138	-0.103	-0.196	-0.376^**^	-0.296^**^	-0.126	-0.150	-0.022	-0.160
HAMD total score	-0.427^**^	-0.242^*^	-0.159	-0.248^*^	-0.020	-0.050	0.071	0.016	-0.012	-0.107	0.070	-0.033
HAMA total score	-0.268^*^	-0.154	-0.045	-0.123	-0.116	-0.040	-0.068	-0.074	-0.131	-0.156	-0.047	-0.135
YMRS total score	0.131	0.144	0.268^*^	0.238^*^	-0.561^**^	-0.394^**^	-0.257^*^	-0.407^**^	0.076	0.107	0.047	0.098
SDS												
Work/study	-0.186	-0.219	-0.287^**^	-0.285^*^	-0.094	-0.167	-0.221	-0.220	0.012	-0.182	-0.123	-0.164
Social life	-0.186	-0.263^*^	-0.323^**^	-0.324^**^	-0.176	-0.166	-0.162	-0.195	0.019	-0.123	-0.129	-0.130
Family life	-0.097	-0.178	-0.199	-0.203	-0.093	-0.143	-0.207	-0.200	0.038	-0.114	-0.064	-0.092

^⁎^Correlations are significant at the 0.05 level (2-tailed). ^⁎⁎^Correlations are significant at the 0.01 level (2-tailed). AFT, Animal Fluency Test; DSST, Digit Symbol Substitution Test; TMT, Trail Making Test. HAMD, The 17-item Hamilton Depression Rating Scale; HAMA, Hamilton Anxiety Rating Scale; YMRS, Young Mania Rating Scale; SDS, the Sheehan Disability Scale.

Multiple regression analysis was performed to clarify the relationships between B-CATS domain scores and demographic and clinical characteristics. The results revealed that in the depression group, the HAMD score was negatively correlated with the AFT score (*R^2^* = 0.548, *F* = 9.424, *p* < 0.001, *β*_HAMD_ = -0.249, *p* = 0.013), and the SDS social life score was negatively correlated with the TMT score (*R^2^* = 0.618, *F* = 13.795, *p* < 0.001, *β*_SDS- Social life_ = -3.811, *p* = 0.017) and total score (*R^2^ =* 0.640, *F* = 10.987, *p* < 0.001, *β*_SDS- Social life_ = -2.035, *p* = 0.030). Furthermore, the YMRS score positively impacted the TMT scores of the patients in the depression group. In the manic group, manic symptoms were negatively correlated with the AFT (*R^2^* = 0.315, *F* = 34.919, *p* < 0.001, *β*_YMRS_ = -0.470, *p* < 0.001), DSST (*R^2^* = 0.204, *F* = 9.606, *p* < 0.001, *β*_YMRS_ = -1.107, *p* < 0.001), and total scores (*R^2^* = 0.390, *F* = 7.570, *p* < 0.001, *β*_HAMD_= -2.055, *p* < 0.001). The current use of psychiatric medications was negatively correlated with the TMT score and total score, and the effect size was substantial. For euthymic patients, only education level had a statistically significant positive effect on the TMT score (*R^2^ =* 0.266, *F*=6.805, *P*<0.001, *β*_education_ = 1.109, *p* < 0.021). The multiple regression analysis results are shown in **Tables 4**, **5** and **6**.

**Table 4:** Multivariate regression analysis of factors influencing the B-CATS score of patients with depression.

Dependent variable	Independent variable	Coefficient	Standardized coefficient	t	P
AFT (*R^2^* = 0.548, *F* = 9.424, p < 0.001)	HAMD ^**^	-0.249	-0.254	-2.541	0.013
DSST (*R^2^* = 0.352, *F* = 4.829 *p* < 0.001)	Marital status^**^	-6.746	-0.330	-2.076	0.041
	Type^**^	5.177	0.237	2.154	0.035
-TMT (*R^2^* = 0.618, *F* = 13.795, *p* < 0.001)	Age at onset^**^	-1.599	-0.769	-2.637	0.010
	YMRS^*^	4.413	0.179	2.403	0.019
	SDS- Social life^**^	-3.811	-0.385	-2.438	0.017
Total score (*R^2^* = 0.640, *F* = 10.987, *p* < 0.001)	SDS- Social life^**^	-2.035	-0.206	2.834	0.030

* Correlations are significant at the 0.05 level (2-tailed). ** Correlations are significant at the 0.01 level (2-tailed). AFT: Animal Fluency Test; DSST: Digit Symbol Substitution Test; TMT: Trail Making Test. HAMD: The 17-item Hamilton Depression Rating Scale; YMRS: Young Mania Rating Scale. SDS: the Sheehan Disability Scale.

**Table 5:** Multivariate regression analysis of factors influencing the B-CATS score of manic or hypomanic individuals.

Dependent variable	Independent variable	Coefficient	Standardized coefficient	t	P
AFT (*R^2^* = 0.315, *F* = 34.919, *p* < 0.001)	YMRS^**^	-0.470	-0.561	-5.909	<0.001
DSST (*R^2^* = 0.204, *F* = 9.606, *p* < 0.001)	Marital status^**^	-5.035	-0.221	-2.135	0.036
	YMRS^**^	-1.107	-0.376	-3.640	<0.001
-TMT (*R^2^* = 0.359, *F* = 6.619, *p* < 0.001)	Age^**^	-0.495	-0.300	-2.218	0.030
	No current drugs^**^	12.645	0.364	3.698	<0.001
Total score (*R^2^* = 0.390, *F* = 7.570, *p* < 0.001)	No current drugs^**^	19.160	0.325	3.385	0.001
	YMRS^**^	-2.055	-0.322	-3.366	0.001

AFT, Animal Fluency Test; DSST, Digit Symbol Substitution Test; TMT, Trail Making Test. YMRS, Young Mania Rating Scale.

* Correlations are significant at the 0.05 level (2-tailed). ** Correlations are significant at the 0.01 level (2-tailed).

**Table 6:** Multivariate regression analysis of factors influencing the B-CATS score of patients in remission.

Dependent variable	Independent variable	Coefficient	Standardized coefficient	t	P
AFT (*R^2^* = 0.267, *F* = 6.845, *p* < 0.001))	–	–	–	–	–
DSST (*R^2^* = 0.209, *F* = 6.682, *p* < 0.001)	–	–	–	–	–
-TMT (*R^2^* = 0.266, *F* = 6.805, *p* < 0.001)	Education^*^	1.109	0.236	2.356	0.021
Total score (*R^2^* = 0.390, *F* = 7.570, *p* < 0.001)	–	–	–	–	–

AFT, Animal Fluency Test; DSST, Digit Symbol Substitution Test; TMT, Trail Making Test.

* Correlations are significant at the 0.05 level (2-tailed). ** Correlations are significant at the 0.01 level (2-tailed).

## 3 Discussion

In this study, we aimed to compare the neurocognitive profiles of patients with BD and HCs using the B-CATS and related normative data for the population. Our findings revealed that patients with BD exhibited significantly poorer performance across all cognitive dimensions measured by the B-CATS compared to HCs, consistent with previous reviews highlighting cognitive impairments in BD (25, 32). Specifically, patients in the depressive phase showed the most severe neurocognitive deficits, while those in the manic or hypomanic phase also demonstrated significant impairments, albeit to a lesser extent. While the results are different from the findings of Ciftci et al (33), who reported uniformly reduced cognitive function across all phases compared to healthy controls, with no significant differences between the three patient groups, possibly because of the differences in population and cognitive assessment tools. Notably, cognitive deficits persisted even in the euthymic phase, though they were less pronounced than during acute episodes. These results confirm that the B-CATS is an effective tool for assessing neurocognitive impairments in BD patients, aligning with its established ability to differentiate between individuals with and without cognitive dysfunction (16, 22). Overall, our study underscores the pervasive nature of cognitive deficits in BD across different illness phases and supports the utility of the B-CATS in capturing these impairments.

Compared with the other three groups, the depression group had the lowest AFT, TMT-A and B-CATS total scores, with significantly lower scores. Depressive episodes are often characterized by cognitive rigidity and slower cognitive processing speed, where patients exhibit difficulties in switching between tasks or thoughts, which impairs their ability to generate words and negatively affects their TMT scores (34–37). Our results revealed that depressive symptoms were negatively correlated with the AFT, DSST, and total B-CATS scores, indicating that higher levels of depressive symptoms are associated with worse neurocognitive performance. This finding is consistent with previous research showing that neurocognitive impairment is a prominent feature of depressive episodes in BD patients, particularly affecting areas such as processing speed, attention, verbal fluency, and executive function (38, 39). Additionally, BD type was found to have an effect on neurocognitive functioning in the depression group, with patients with BD type I exhibiting worse neurocognitive performance than those with BD type II. This aligns with previous studies suggesting that BD type I, which is typically associated with more severe and frequent mood episodes, may lead to greater neurocognitive deficits over time (40) (41). However, the differences in cognitive function between patients with BD type I and type II are controversial (42, 43). Furthermore, factors such as age, age at illness onset, duration of illness, and attack frequency were negatively correlated with neurocognitive scores across all B-CATS domains, suggesting that a longer disease duration and an earlier onset may contribute to more significant neurocognitive decline in BD patients. This finding underscores the chronic nature of neurocognitive dysfunction in BD patients and the potential cumulative effect of recurrent mood episodes on neurocognitive health. Interestingly, employment status was positively correlated with B-CATS scores (except for the DSST score), suggesting that social engagement and work participation may help preserve neurocognitive function in BD patients. This finding is consistent with research indicating that higher levels of social functioning and activity are associated with better neurocognitive performance and overall mental health outcomes (44).

In the mania group, manic symptoms were inversely correlated with the B-CATS domain scores and total score, indicating that increased manic symptoms are associated with worse neurocognitive performance. This finding is consistent with findings that mania impairs executive function, attention, and memory, with patients often exhibiting heightened impulsivity and neurocognitive disorganization (45). Moreover, psychiatric medication use was found to be associated with lower B-CATS total scores and TMT scores, suggesting that current medication use may have a negative impact on neurocognitive functioning. This result may reflect the side effects of medications commonly used to treat BD, such as antipsychotics, which have been associated with neurocognitive slowing and impairments in processing speed and executive function. However, the impact of pharmacological treatments on neurocognitive function is mixed, with numerous studies reporting conflicting results (46–48). These findings underscore the need for clinicians to carefully weigh the benefits and risks of medications when treating BD patients, particularly in terms of their cognitive effects. Future studies should explore neurocognitive assessments both before and after the initiation of treatment, although this would require participants to be emotionally stable prior to starting psychiatric medication, which is a relatively rare clinical condition. In the present study, we observed that there were no statistically significant differences in the DSST scores between the mania/hypomania group and the depression group. This finding may seem unexpected given the well-established neurocognitive differences between the two phases in BD patients, as Martinez et al (38). have consistently reported distinct cognitive profiles in these phases. The complexity and sensitivity of the DSST and the presence of shared neurocognitive dysfunction may explain the lack of significant differences in the DSST scores between the mania and depression groups. For example, both manic and depressive episodes are associated with impairments in attention and processing speed, which are key components of the DSST. This overlap in cognitive deficits may have obscured phase-specific differences in our study.

As shown in this study, neurocognitive dysfunction in BD patients during remission—while less severe than that during active mood episodes—remains a significant concern. As demonstrated by numerous previous studies, patients with BD still exhibit significant cognitive impairment during the remission phase, which severely impacts their quality of life and social functioning (49–51). The reasons for this persistent neurocognitive impairment, despite the resolution of mood symptoms, are multifactorial and related to both biological and psychosocial factors (32, 52). Even when mood symptoms subside, the neurochemical dysregulation underlying BD can still impact neurocognitive performance. Chronic neurochemical imbalances in BD patients, especially in the dopamine, serotonin, and glutamate systems, may continue to affect neurocognitive function during remission (53–55). Dopamine plays a key role in attention, working memory, and executive function. Glutamate is also involved in synaptic plasticity and memory formation. This study suggested that neurocognitive function in BD patients in the remission phase is associated with age, education level, age at illness onset, and disease duration. These findings suggest that higher education may act as a cognitive reserve, potentially mitigating the impact of BD-related cognitive impairments. Clinically, this highlights the importance of early educational interventions and cognitive training programs for BD patients, particularly those with lower educational attainment, to enhance their cognitive resilience and improve long-term outcomes. However, after multiple regression analysis, only education level remained clinically significant. Given that previous studies have also indicated a lack of sufficient evidence for progressive neurocognitive decline in BD patients, education level serves as an indicator of knowledge reserves, which have a protective effect on the maintenance of neurocognitive function (56, 57). However, neurocognitive dysfunction during euthymia further emphasizes that mood stabilization does not necessarily equate to full neurocognitive recovery, which may be influenced by the chronic course of BD, recurrent episodes, and side effects of pharmacological treatment.

Neurocognitive dysfunction in BD may arise from widespread abnormalities across large-scale brain networks. Meta-analyses integrating resting-state functional connectivity (rs-FC) and voxel-based morphometry (VBM) have demonstrated that BD is characterized by hypoconnectivity within the default mode network (DMN), and hyperconnectivity within the affective network (AN) and ventral attention network (VAN) (58–60). The frontoparietal network (FPN) shows both hypo- and hyperconnectivity across subregions, and disrupted between-network connectivity—particularly involving the thalamic network (TN)—further indicates impaired functional integration across cognitive and emotional processing systems (58, 61). These functional disruptions are often accompanied by reductions in grey matter volume in regions such as the insula, inferior frontal gyrus, and anterior cingulate cortex, which are key hubs for emotion regulation and executive control (60, 62). Task-based functional MRI studies further support the concept of network-level dysfunction, showing that cognitively impaired individuals with BD exhibit reduced activation in the dorsolateral prefrontal cortex (DLPFC) and frontoparietal regions within the cognitive control network (CCN), along with increased activation in the DMN during working memory tasks. This pattern—characterized by insufficient recruitment of task-relevant regions and a failure to suppress task-irrelevant DMN activity—may represent a core neural mechanism underlying executive dysfunction in BD (63, 64).

Notably, B-CATS demonstrates distinct advantages in efficiency and practicality compared to other cognitive assessment instruments. In contrast to the MCCB, which requires 60–90 minutes to assess multiple cognitive domains, the B-CATS provides a rapid 10-minute global cognitive screening through subtests such as the TMT A/B, DSST, and AVF. This brevity minimizes patient fatigue and clinical administration burden, particularly advantageous for longitudinal monitoring of cognitive trajectories in BD, where deficits often persist during euthymic phases. The tool effectively captures subtle cognitive impairments characteristic of BD. Psychometrically, the B-CATS exhibits acceptable internal consistency (Cronbach’s α = 0.6) (22), while maintaining strong convergent validity with the MCCB total score (r = 0.76, p < 0.01) (21), supporting its ecological validity. Its administration simplicity enables use by non-specialists, enhancing accessibility in resource-constrained settings. The Cronbach's alpha of the B-CATS was 0.77 for this study, which was acceptable. This result indicated that its subtests (e.g., AFT, DSST, -TMT) reliably measure the same underlying construct of cognitive function in bipolar disorder. While slightly below the ideal threshold of 0.80, this value supports the tool’s usability for group-level comparisons in clinical research. However, its limitation lies in the inability to delineate domain-specific deficits (e.g., social cognition), necessitating supplementary assessments such as the MCCB for comprehensive profiling. Overall, the B-CATS serves as a pragmatic, reliable screening instrument for clinical decision-making, though multidimensional evaluations remain essential for complex cases.2 Limitations and strengths

Several limitations of this study must be addressed, and caution is necessary when interpreting the results. First, this was a cross-sectional descriptive study, as are most investigations of neurocognitive function. Because BD is a chronic illness involving not only multiple episodes but also fluctuating residual symptoms, conducting a respective survey to assess neurocognitive function over extended periods is vital. Second, sociodemographic and clinical data were collected via recall assessment and thus may be affected by memory. The neurocognitive test results were assessed by a single rater. Different aspects of the assessment should ideally be implemented by different researchers who are blinded to each other’s assessment results. Although we applied the B-CATS to assess neurocognitive function in BD patients and the findings offer preliminary support for the applicability of B-CATS in assessing cognitive function in patients with bipolar disorder in China, the reliability and validity of the B-CATS in this patient population have not been validated. The absence of standardized normative data limits the generalizability of the results. Future studies should aim to establish the psychometric properties of the B-CATS specifically for BD populations in China. Such efforts are essential for establishing the tool’s reliability and validity across diverse populations and for enhancing cross-cultural comparability in cognitive research on bipolar disorder. A third limitation of the present study is the sample size, which might have decreased the statistical power. A larger sample size would have allowed the use of more complex regression models to explore the impact of a wider range of clinical and psychological factors on B-CATS domain scores. Moreover, we included a mixed sample of inpatients and outpatients with different clinical states. Factors related to acute hospitalization, such as the severity of symptoms and the effects of intensive treatment, as well as mood symptoms, may have a nonnegligible effect on neurocognitive functioning. Future studies should consider stratifying analyses based on hospitalization status to better control for these confounding factors. Another notable limitation of the present study is the lack of detailed information on psychotropic medication use. While we recorded whether participants were taking psychiatric medications at the time of assessment, we did not further categorize the type of medication (e.g., lithium, anticonvulsants, antipsychotics), dosage, or treatment duration. This limitation precluded us from evaluating the potential differential effects of specific pharmacological agents on cognitive performance. Given the known cognitive effects of certain medications commonly used in bipolar disorder, future studies should incorporate more comprehensive medication data to clarify their impact on cognitive functioning across illness phases. Despite these limitations, the strengths of our study include the relatively large sample size and the use of four comparison groups (hypomanic/manic, depressed, euthymic, and healthy groups), which have been better characterized in previous studies. Furthermore, the B-CATS is a quick and simple tool for assessing neurocognitive function. We attempted to apply this tool to a population in different phases of BD, thereby contributing to the expansion of research in this area to some extent.

## 4 Conclusion

In conclusion, this cross-sectional sample of euthymic, hypomanic or manic and depressed BD patients presented neurocognitive deficits in relation to HCs, supporting its potential role as a trait marker of BD. The evidence suggests that even during euthymic states, when mood symptoms are controlled, BD patients often exhibit persistent neurocognitive impairments, particularly in areas such as executive function, attention, and working memory. In patients with acute BD, neurocognitive function is negatively correlated with the severity of mood symptoms, meaning that the more severe the depressive or manic symptoms are, the poorer the neurocognitive function. The observed amplified group differences during acute episodes may further reflect a state-dependent modification, possibly linked to mood episode severity or neurobiological changes during illness exacerbation. Future longitudinal studies tracking within-individual BCAT fluctuations across mood states, alongside mechanistic investigations (e.g., neuroimaging or inflammatory markers), are needed to disentangle these contributions. Controlling for confounders such as medication, illness chronicity, and symptom severity will be critical in refining BCAT’s utility as a biomarker in BD. Additionally, neurocognitive impairment in BD patients is a complex and persistent issue that significantly impacts patients’ quality of life and social functioning. Future research should focus on strategies to mitigate neurocognitive decline, including neurocognitive training, neuroprotective therapies, and individualized treatment plans, to enhance neurocognitive recovery and overall quality of life for individuals with BD.

## Data Availability

The raw data supporting the conclusions of this article will be made available by the authors, without undue reservation.
